# Clinically Suspected Invasive Meningococcal Disease Presenting With Purpura Fulminans‐Like Rash, Septic Shock, Multiorgan Dysfunction, and Recovery From ARDS in a Previously Healthy Child

**DOI:** 10.1002/ccr3.73144

**Published:** 2026-07-10

**Authors:** Shatha Omar, Adil Alsweis, Amr Khaled, Noor Nabresi, Mosub Qatu, Yahya Qahwash, Mahfouth Jallad

**Affiliations:** ^1^ Department of Medicine, Faculty of Medicine and Health Sciences An‐Najah National University Nablus Palestine; ^2^ Department of Pediatrics Rafidia Hospital Nablus Palestine

**Keywords:** disseminated intravascular coagulation, invasive meningococcal disease, *Neisseria meningitides*, purpura fulminans, septic shock

## Abstract

Clinically suspected invasive meningococcal disease can deteriorate rapidly in previously well children. Prompt recognition of the petechial–purpuric rash with shock, early empirical antibiotics, and aggressive supportive pediatric intensive care are cornerstones of management as multiorgan dysfunction and Acute Respiratory Distress Syndrome may occur, but they are reversible with appropriate care.

**Abbreviations**
AKI: acute kidney injury
ARDS: acute respiratory distress syndrome
BG: blood gas
BP: blood pressure
CSF: cerebrospinal fluid
DIC: disseminated intravascular coagulation
ED: emergency department
GCS: Glasgow Coma Scale
HIV: human immunodeficiency virus
HPF: high‐power field
HR: heart rate
ICU: intensive care unit
IMD: invasive meningococcal disease
IV: intravenous
MV: mechanical ventilation

*N. meningitidis*

:
*Neisseria meningitidis*

PaO_2_/FiO_2_
: ratio of arterial oxygen partial pressure to fractional inspired oxygen
PCR: polymerase chain reaction
PEEP: positive end‐expiratory pressure
PICU: pediatric intensive care unit
PPE: personal protective equipment
RR: respiratory rate
SIMV: synchronized intermittent mandatory ventilation
SpO_2_
: peripheral oxygen saturation

## 1 Introduction

*Neisseria meningitidis* ( *N. meningitidis* ) is an encapsulated, aerobic, Gram‐negative diplococcus [1]. It is an obligate human pathogen that frequently colonizes the nasopharynx but can cause invasive meningococcal disease (IMD), including meningitis and septicemia, after entering the bloodstream and crossing the blood–brain barrier [2]. Approximately 10% of the global population are asymptomatic carriers [1].

Meningitis is characterized by fever, headache, and neck stiffness. Meningococcemia may present with petechiae, purpura, limb pain, cold extremities, and fulminant sepsis. These conditions are the most common presentations of IMD and may coexist, especially in children and adolescents [3]. The most serious form of meningococcal septicemia is fulminant meningococcemia, which is marked by a high fatality rate, rapid onset, septic shock, and purpura fulminans [4].

Early symptoms may be nonspecific, including abdominal pain, diarrhea, gastroenteritis, and peritonitis, which can delay diagnosis and treatment [5]. Several conditions increase the risk of acquiring IMD, including asplenia, human immunodeficiency virus (HIV) infection, complement deficiency, use of complement inhibitor drugs such as eculizumab, military service, and occupational exposure among microbiologists working with *N. meningitidis* [3].

Diagnosis is based on clinical findings and confirmed by laboratory investigations such as blood culture, cerebrospinal fluid (CSF) analysis, and polymerase chain reaction (PCR), which can detect the organism even after antibiotic therapy and identify serogroups for epidemiological purposes [5]. Mortality remains 10%–15%, mainly due to multiorgan failure and septic shock [4].

This case describes a previously healthy 12‐year‐old child who rapidly developed progressive IMD complicated by septic shock, coagulopathy, acute kidney injury, and acute respiratory distress syndrome (ARDS). It emphasizes the difficulty of diagnosis and the importance of early intensive care.

## 2 Case History and Examination

A 12‐year‐old male patient was in his usual state of health until 2 days before admission, when he started complaining of high‐grade fever accompanied by vomiting, non‐bloody diarrhea, and neck pain. He gradually became lethargic and weak, with the appearance of a rapidly progressive rash characterized by small spots that turned into dark purpuric patches, as described by his family. There was no significant past medical or surgical history, no known medication allergies, and his vaccinations were up to date without any complications.

The patient appeared toxic, severely ill, hypoactive, and dehydrated upon arrival at the emergency department (ED). Initial vital signs revealed fever, severe tachycardia, severe hypotension, mild tachypnea, and slightly decreased oxygen saturation (Table 1). Due to persistent hypotension, immediate resuscitation was started in the ED with aggressive intravenous (IV) fluid administration and early vasopressor therapy. Empiric broad‐spectrum IV antibiotics were started immediately without delay, including meningitic‐dose ceftriaxone and vancomycin, after obtaining blood cultures, which later showed no growth.

**TABLE 1:** Initial vital signs at admission showed hemodynamic instability.

Parameter	Patient value on admission	Normal range
Temperature	Febrile (fever present)	36.5°C–37.5°C
Heart rate (HR)	150 beats/min	60–100 beats/min
Blood pressure (BP)	80/40 mmHg	~100–120/60–80 mmHg
Respiratory rate (RR)	20s breaths/min	12–20 breaths/min
Oxygen saturation (SpO_2_)	94% on room air	≥ 95%–100%

**FIGURE 1:**
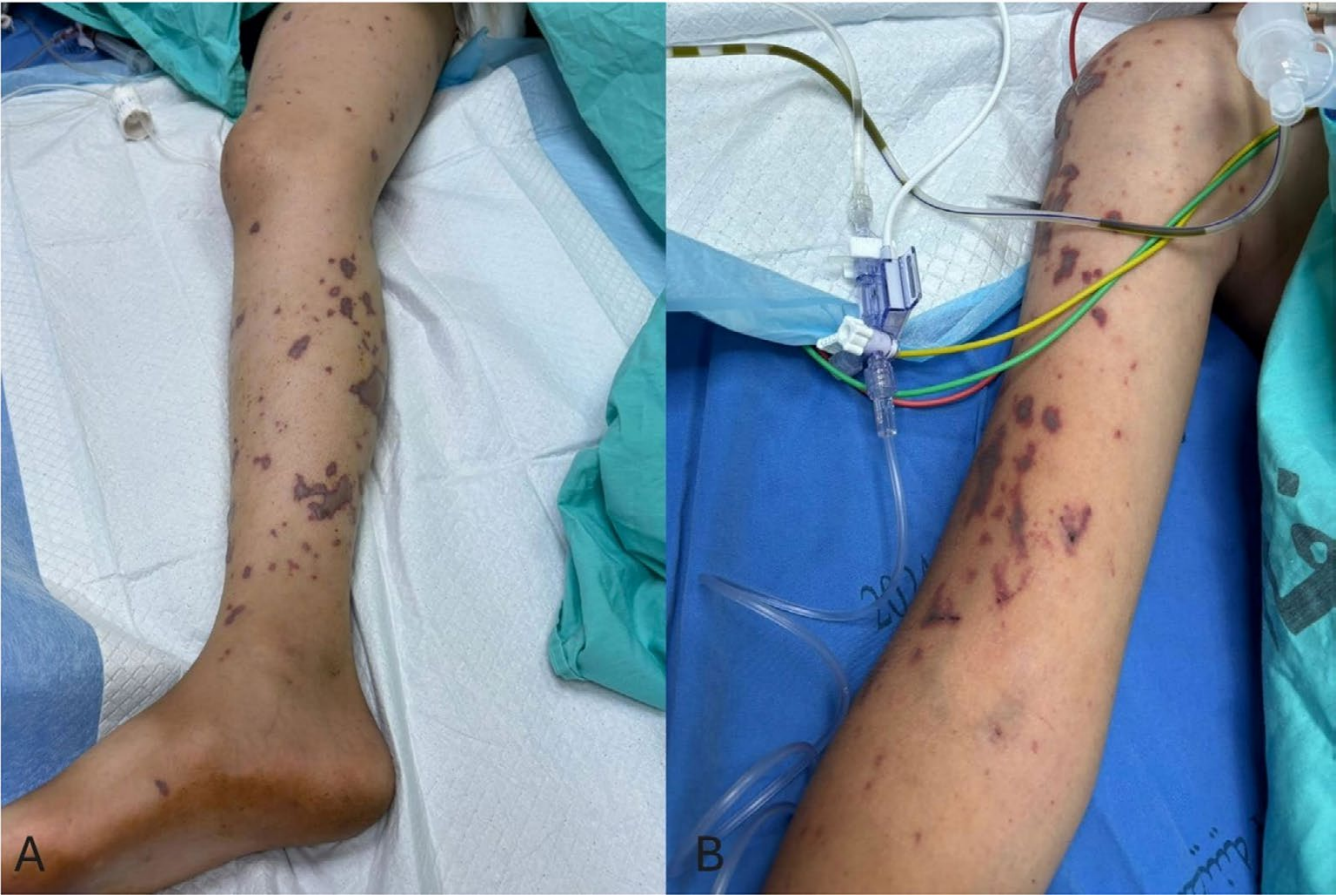
Diffuse petechial and ecchymotic rash involving the body at presentation. (A): Clinical photograph of lower extremity demonstrating scattered erythematous non‐blanching petechiae, purpuric macules, and irregular ecchymotic plaques on the left thigh, knee, and ankle. The plaques have slight dark central discoloration seen in evolving hemorrhagic necrosis/purpura fulminans in the context of septic shock, thrombocytopenia, and coagulopathy. (B) Clinical photograph of the upper extremity from the shoulder to the arm showing multiple non‐blanching petechiae, purpuric macules, and irregular ecchymotic plaques of variable size. Multiple lesions show violaceous‐to‐dark central discoloration, indicating progressive hemorrhagic necrosis.

Skin manifestations observed during ED evaluation included diffuse non‐blanching petechiae and purpura involving the trunk and extremities, with more noticeable involvement of the upper and lower limbs (Figure 1A,B). The lesions ranged from pinpoint petechiae to irregular purpuric macules and ecchymotic patches. Some large violaceous lesions were seen on the upper limb, thighs, legs, and ankles, with some lesions demonstrating dark central discoloration, indicating the development of hemorrhagic necrosis. Overall, the lesions were scattered to confluent and were highly suggestive of purpura fulminans. Neurologic examination revealed significant neck rigidity and positive Kernig and Brudzinski signs. However, he was conscious, and his Glasgow Coma Scale (GCS) score was 13–15 without any focal neurological deficits. Chest examination was initially unremarkable, and no cardiac murmur was heard on cardiac examination.

Infection control policies were implemented as soon as the patient was diagnosed with probable invasive meningococcal disease. Isolation measures, including contact and droplet precautions, in addition to the placement of the patient in an isolation room, were initiated. Healthcare providers used personal protective equipment (PPE), including gloves, gowns, surgical masks, and goggles. N95 masks were used for aerosol‐generating procedures. Equipment was used as dedicated devices whenever possible, and strict disinfection protocols were followed.

## 3 Investigations and Treatment

Initial laboratory studies revealed leukocytosis with neutrophil predominance, severe thrombocytopenia, elevated inflammatory markers, prolonged prothrombin time (PT), partial thromboplastin time (PTT), and international normalized ratio (INR), severe metabolic and lactic acidosis, as well as acute kidney injury with elevated serum creatinine (Table 2). Urinalysis was also performed and showed significant hematuria (+3 blood, 20–25 RBCs/HPF), with elevated urine sodium (59 mmol/L) and a fractional excretion of sodium (FeNa) of 1%. Renal ultrasound demonstrated mildly increased bilateral renal parenchymal echogenicity without hydronephrosis, consistent with acute kidney injury without obstructive changes. These findings were consistent with septic shock complicated by disseminated intravascular coagulation (DIC) and multiorgan dysfunction.

**TABLE 2:** Initial laboratory findings at admission showed an inflammatory response, coagulopathy, acute kidney injury, and metabolic acidosis.

Test	Admission value	Reference range
WBC	21 × 10^3^/μL	4.5–13 × 10^3^/μL
Neutrophils	92%	40%–70%
Hemoglobin	12 g/dL	11.5–15.5 g/dL
MCV	82 fL	80–96 fL
Platelets	86 × 10^3^/μL	150–450 × 10^3^/μL
ESR	10 mm/h	0–20 mm/h
CRP	218 mg/L	< 5 mg/L
Potassium (K)	3.9 mmol/L	3.5–5.0 mmol/L
Sodium (Na)	140 mmol/L	135–145 mmol/L
Calcium (Ca)	7 mg/dL	8.5–10.5 mg/dL
PT	28 s	11–14 s
PTT	64 s	25–35 s
INR	2.4	0.8–1.2
AST	37 U/L	10–40 U/L
ALT	16 U/L	7–35 U/L
Albumin	2.4 g/dL	3.5–5.0 g/dL
Creatinine	3.7 mg/dL	0.4–0.9 mg/dL
BUN	33 mg/dL	7–20 mg/dL
pH	7.2	7.35–7.45
HCO_3_^−^	13.1 mmol/L	22–26 mmol/L
Lactate	8 mmol/L	0.5–2 mmol/L

Abbreviations: ALT, alanine aminotransferase; AST, aspartate aminotransferase; BUN, blood urea nitrogen; CRP, C‐reactive protein; ESR, erythrocyte sedimentation rate; HCO_3_^−^, bicarbonate; INR, international normalized ratio; MCV, mean corpuscular volume; PT, prothrombin time; PTT, partial thromboplastin time; WBC, white blood cell.

Due to the severity of his clinical condition and hemodynamic instability, the patient was admitted to the Pediatric Intensive Care Unit (PICU) under isolation measures. Supportive intensive care measures included head elevation, regular neurological checks, continuous cardiorespiratory monitoring, and strict input/output recording with Foley catheter insertion. PCR testing was not performed because lumbar puncture was deferred due to hemodynamic instability, severe thrombocytopenia, and significant coagulopathy; therefore, no CSF sample was safely obtained for PCR analysis. In addition, blood‐based meningococcal PCR testing was not available at our institution at the time of presentation. As a result, the diagnosis remained clinically suspected rather than microbiologically confirmed.

Supportive hemodynamic management was continued with IV fluids and norepinephrine administration through a femoral venous line placed under ultrasound guidance. Antibiotic therapy was continued with ceftriaxone and vancomycin. Dexamethasone was also administered as adjunctive therapy to reduce inflammation and the risk of neurologic complications. Because of DIC, fresh frozen plasma and vitamin K were given, with serial monitoring of coagulation profiles. The patient showed partial hemodynamic and metabolic improvement during the first 24–48 h of admission, but he remained critically ill, with evidence of ongoing systemic inflammation, hematological abnormalities, vasopressor dependence, and progressive multiorgan dysfunction. During the ICU stay, infectious disease consultation raised concern for possible rickettsial infection because of the vasculitic nature of the rash, and doxycycline was added.

**FIGURE 2:**
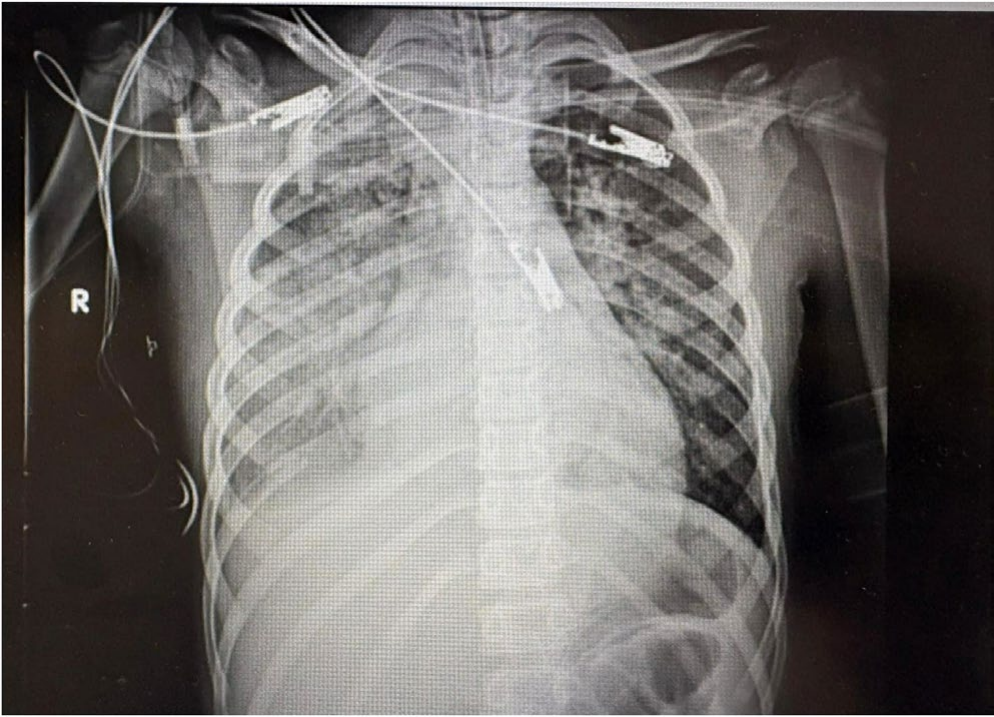
Chest X‐ray after development of respiratory distress showing bilateral pulmonary infiltrates, more pronounced on the right. Portable anteroposterior chest radiograph obtained after acute respiratory deterioration demonstrating bilateral patchy air‐space/interstitial opacities, greater in the right lung, particularly in the right upper and mid‐lung zones, with milder left perihilar and lower‐zone opacities. The findings are consistent with bilateral pulmonary infiltrates/consolidative changes in the setting of worsening hypoxemia and suspected sepsis‐associated acute respiratory distress syndrome.

The hospital course was further complicated on the third day of hospitalization by acute respiratory deterioration with hypoxia. A portable anteroposterior chest X‐ray demonstrated new bilateral patchy interstitial opacities, more pronounced in the right lung, with additional milder left perihilar and lower‐zone opacities, prompting initiation of meropenem and discontinuation of ceftriaxone (Figure 2). Subsequently, chest ultrasound demonstrated right‐sided consolidation with pleural effusion in the setting of evolving ARDS. The patient required endotracheal intubation and mechanical ventilation with synchronized intermittent mandatory ventilation (SIMV) pressure‐control mode and high positive end‐expiratory pressure (PEEP), along with continuous sedation and analgesia.

Regarding the new bilateral pulmonary opacities, sputum culture was not obtained because no adequate respiratory specimen was available before intubation. After intubation, endotracheal aspirate was not collected because there were no adequate purulent respiratory secretions, and the patient had already received broad‐spectrum antibiotics, which likely reduced the diagnostic yield of respiratory cultures. Bronchoalveolar lavage was not pursued because of the patient's critical condition, severe hypoxemia, thrombocytopenia, and coagulopathy. Clinically and radiographically, the respiratory deterioration was considered more consistent with sepsis‐associated ARDS than primary bacterial pneumonia, particularly in the setting of septic shock, systemic inflammation, bilateral pulmonary opacities, and subsequent improvement with ventilatory and intensive supportive management.

## 4 Outcome and Follow‐Up

**FIGURE 3:**
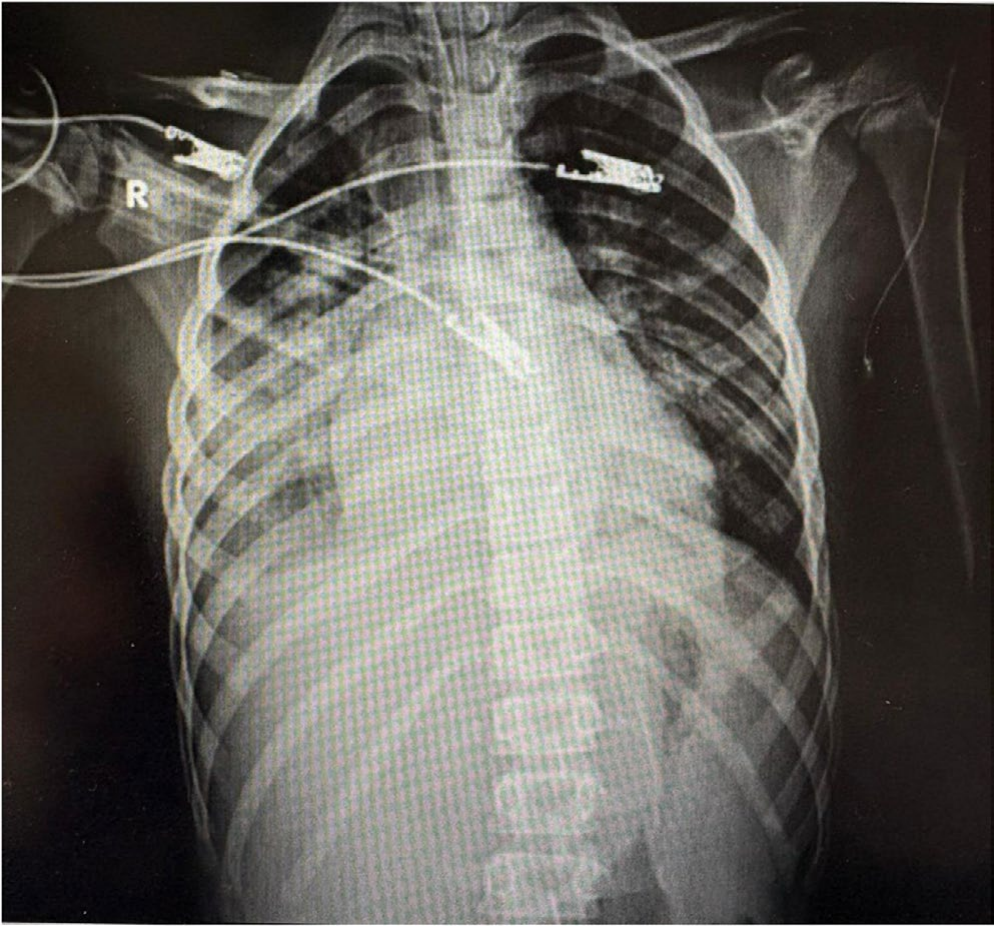
Follow‐up chest X‐ray after 2 days of mechanical ventilation showed an interval improvement of bilateral pulmonary infiltrates. Portable anteroposterior chest radiograph after 2 days of mechanical ventilation showed partial interval resolution of bilateral air‐space/interstitial opacities previously identified. Patchy opacities are predominantly in the right upper and mid‐lung zones, with mild residual left‐sided haziness. Findings are consistent with radiographic improvement of ARDS/acute respiratory failure with supportive ventilatory management.

After 2 days of mechanical ventilation and intensive supportive management, follow‐up portable anteroposterior chest radiography showed interval improvement in the previously noted bilateral pulmonary opacities (Figure 3). These radiographic findings were consistent with partial improvement of the patient's acute respiratory distress. Serial laboratory follow‐up revealed improvement in renal function, lactate level, metabolic acidosis, and coagulation parameters, while leukocytosis, thrombocytopenia, and systemic inflammation persisted (Table 3). Transient elevation of troponin and creatine kinase (CK) was noted during the critical phase of septic shock and subsequently declined. In the absence of arrhythmias, ischemic electrocardiographic changes, chest pain, or significant structural cardiac abnormalities, these findings were interpreted as secondary to sepsis‐related myocardial stress and increased physiologic demand rather than primary myocardial disease (Table 3).

**TABLE 3:** Serial laboratory follow‐up during hospitalization showing trends in inflammation, coagulation, renal function, blood gases, and cardiac biomarkers.

Test	On the 2nd day of admission	On the 3rd day of admission	Reference range
WBC	23.2 × 10^9^/L	35 × 10^9^/L	4.5–13 × 10^3^/μL
Neutrophils/ANC	ANC 20.8 × 10^9^/L	ANC 31.1 × 10^9^/L	40%–70%
Hemoglobin	9.4 g/dL	9 g/dL	11.5–15.5 g/dL
Platelets	43 × 10^9^/L	52 × 10^9^/L	150–450 × 10^3^/μL
CRP	373 mg/L then 358 mg/L	253 mg/L, then improving	< 5 mg/L
ESR	75 mm/h	75–100 mm/h	0–20 mm/h
Creatinine	1.44 then 1.16 mg/dL	0.85–0.9 mg/dL	0.4–0.9 mg/dL
BUN	31 then 29 mg/dL	29–40 mg/dL	7–20 mg/dL
Lactate	2.42 mmol/L	~1.5 mmol/L	0.5–2 mmol/L
pH	7.46, then 7.53 during respiratory distress on the 3rd day	7.32–7.37 on MV	7.35–7.45
Bicarbonate	22.6 mmol/L	~22 mmol/L	22–26 mmol/L
PT	15.9 s	15.4 s	11–14 s
PTT	36 s	24 s	25–35 s
INR	1.26	1.2	0.8–1.2
D‐dimer	> 20,000	—	< 500 ng/mL (FEU)
Fibrinogen	675	—	200–400 mg/dL
Troponin	1.657	0.86 then improving	< 0.04 ng/mL
CK	1132	593 then improving	26–192 U/L
Sodium	145 mmol/L	149 mmol/L	135–145 mmol/L
Potassium	3.7 mmol/L	4 mmol/L	3.5–5 mmol/L
Calcium	8.3 mg/dL	8 mg/dL	8.5–10.5 mg/dL
Albumin	3.2 g/dL	3.2 g/dL	3.5–5.0 g/dL

Abbreviations: ANC, absolute neutrophil count; BUN, blood urea nitrogen; CBC, complete blood count; CK, creatine kinase; CRP, C‐reactive protein; ESR, erythrocyte sedimentation rate; FEU, fibrinogen equivalent units; INR, international normalized ratio; MV, mechanical ventilation; PT, prothrombin time; PTT, partial thromboplastin time; WBC, white blood cell.

Although partial stabilization of some organ functions was achieved, the patient remained in a life‐threatening condition requiring mechanical ventilatory support. Considering the severity of his illness, transfer to a tertiary care center was recommended for further advanced management under the supervision of pediatric intensive care specialists and a nephrologist. At the tertiary care facility, repeat cardiac evaluation with echocardiography showed no significant structural abnormality and demonstrated low‐normal systolic function, with an ejection fraction within the normal age‐appropriate range. Repeat blood cultures also remained negative.

The patient subsequently showed gradual clinical improvement with recovery from ARDS, allowing successful weaning from mechanical ventilation and progressive overall clinical stabilization. He was then transferred from the intensive care unit to the general ward. His clinical condition improved, but he was maintained on intravenous meropenem and ceftriaxone to complete therapy, and dermatology follow‐up was recommended for persistent skin lesions.

The patient was eventually discharged from the hospital after 2 weeks in good general condition, active, and without medication, while continuing outpatient dermatology follow‐up for skin lesion care. Table 4 summarizes the timeline of major clinical events from initial presentation, early intensive care unit management, respiratory failure, transfer, to the tertiary center, and ultimate clinical recovery.

**TABLE 4:** Clinical timeline from presentation to discharge.

Hospital phase	Main events
Presentation	Fever, severe neck pain, vomiting, non‐bloody diarrhea, toxic appearance, shock, petechial–ecchymotic rash, and meningeal signs
Initial ICU admission	Suspected meningococcemia/meningitis with septic shock, acute kidney injury, metabolic acidosis, thrombocytopenia, and coagulopathy
Early management	Isolation, intravenous fluids, norepinephrine, ceftriaxone, vancomycin, dexamethasone, vitamin K, fresh frozen plasma, and close neurological and hemodynamic monitoring
First 24–48 h	Hemodynamic and renal improvement, with persistent toxicity, thrombocytopenia, elevated inflammatory markers, and persistent meningeal signs
Infectious disease input	Doxycycline was added to cover possible rickettsial infection while continuing treatment for suspected meningococcemia
Respiratory deterioration	Tachypnea, desaturation, bilateral pulmonary infiltrates more pronounced on the right, pleural effusion/consolidation, and suspected ARDS
Later ICU course	Intubation, mechanical ventilation, sedation, escalation to meropenem, continued vasopressor support, and supportive care
Tertiary center course and outcome	Gradual clinical improvement, recovery from ARDS, successful weaning from mechanical ventilation, transfer to the general ward, and discharge in good general condition with outpatient dermatology follow‐up

## 5 Discussion

Meningococcemia is caused by bloodstream invasion with *N. meningitidis* and may present as septicemia, meningitis, or a combination of both; adolescents represent one of the recognized vulnerable age groups [6]. This case illustrates a fulminant pediatric presentation of clinically suspected invasive meningococcal disease complicated by septic shock, coagulopathy, acute kidney injury, transient sepsis‐related myocardial stress, and ARDS. The initial presentation was highly concerning because the patient was previously healthy and presented with fever, severe neck pain, vomiting, diarrhea, toxic appearance, hypotension, altered activity, positive meningeal signs, and a diffuse non‐blanching petechial–purpuric and ecchymotic rash. Because invasive meningococcal disease can progress rapidly from early nonspecific symptoms to septic shock and multiorgan dysfunction, empirical antimicrobial treatment should be instituted immediately when meningococcal disease is suspected [7].

The patient's instability and coagulopathy limited the diagnostic workup, and the diagnosis remained clinical rather than microbiologically confirmed. Blood cultures obtained at our hospital and at the tertiary center showed no growth, and lumbar puncture was not performed because the patient had shock, thrombocytopenia, and abnormal coagulation parameters. Pediatric guidance emphasizes that antimicrobials should not be delayed in an unwell child with suspected meningitis or sepsis, and lumbar puncture should be deferred in the presence of cardiovascular compromise or coagulopathy [8, 9]. CDC diagnostic guidance indicates that invasive *N. meningitidis* disease is clinically diagnosed, while culture and nucleic acid amplification testing, including PCR, may be used for confirmation [10]. Culture may have decreased sensitivity if specimens are not properly handled or if they are obtained after antimicrobial therapy has commenced [10].

An important aspect of this case was early hematologic involvement, including thrombocytopenia, prolonged PT/PTT, increased INR, and markedly increased D‐dimer, findings compatible with sepsis‐associated coagulopathy and presumptive DIC. The petechial and ecchymotic rash likely reflected the combined effects of thrombocytopenia, endothelial injury, microvascular thrombosis, and consumptive coagulopathy. Purpura fulminans is a severe manifestation of this process and is classically described as a life‐threatening syndrome marked by DIC and endovascular thrombosis, producing characteristic cutaneous purpura [11]. Although frank skin necrosis or limb ischemia was not documented, the presence of diffuse petechial–ecchymotic rash, shock, thrombocytopenia, and coagulopathy placed this patient on the severe end of the meningococcal sepsis spectrum. Supportive correction with fresh frozen plasma and vitamin K was followed by improvement in INR and PT/PTT, while thrombocytopenia persisted during the early critical phase.

Renal involvement was also clinically relevant. The patient developed severe acute kidney injury, with creatinine rising to 3.7 mg/dL in the context of shock, metabolic acidosis, elevated lactate, and abnormal urinalysis. Renal ultrasound showed mildly increased bilateral renal parenchymal echogenicity without hydronephrosis, favoring medical renal involvement rather than obstruction. In the setting of septic shock, the acute kidney injury was most likely multifactorial, related to renal hypoperfusion, inflammatory endothelial injury, and possible acute tubular injury. The subsequent improvement in creatinine to approximately 0.9 mg/dL after resuscitation and hemodynamic support suggests a substantial reversible component, likely driven by shock‐associated renal hypoperfusion. This rapid renal recovery is an important favorable feature compared with more severe cases complicated by renal cortical necrosis or prolonged renal replacement therapy [12].

Transient elevation of cardiac biomarkers was noted during the critical phase of septic shock. Troponin and creatine kinase levels were elevated in the absence of documented arrhythmia, ischemic electrocardiographic changes, chest pain, or significant structural cardiac abnormality. Echocardiography at the tertiary center showed low‐normal systolic function but no major structural abnormality. These findings were therefore considered more consistent with transient sepsis‐related myocardial stress or sepsis‐related myocardial injury rather than primary myocardial disease. Sepsis‐induced myocardial dysfunction is a recognized complication of pediatric sepsis and septic shock, may be clinically silent, and is often reversible with resolution of the septic process [13]. In addition, cardiac troponin I may be elevated in septic children and does not necessarily correlate directly with the degree of myocardial dysfunction [13]. Therefore, the low‐normal systolic function in this patient, together with the absence of significant structural abnormalities, supports a transient sepsis‐related process. Follow‐up cardiac assessment may be considered if symptoms, ventricular dysfunction, or biomarker abnormalities persist.

The respiratory deterioration on the third hospital day was another important complication. The patient developed tachypnea, grunting, nasal flaring, desaturation, bilateral infiltrates, pleural effusion, and eventually respiratory failure requiring intubation and mechanical ventilation. This course was compatible with sepsis‐associated ARDS. The ratio of arterial oxygen partial pressure to fractional inspired oxygen was approximately 172, indicating at least moderate oxygenation impairment. Pediatric acute respiratory distress syndrome is a known complication of sepsis and is associated with considerable morbidity, high intensive care requirements, and an increased risk of death [14]. The patient gradually improved at the tertiary care center and was weaned from mechanical ventilation, suggesting recovery after acute respiratory failure. This improvement is clinically important because ARDS in the setting of septic shock and coagulopathy reflects severe systemic endothelial and inflammatory injury.

Similar cases in the literature support the severity and plausibility of this presentation. Ghosh et al. reported a pediatric case of fatal meningococcal sepsis with subsequent ARDS requiring mechanical ventilation and immune‐mediated complications, including reactive arthritis and thrombocytopenia. Their report highlights that meningococcal disease ranges from occult bacteremia to fatal sepsis and shock with ARDS, and that acute complications may include purpura fulminans, renal failure, myocardial failure, and ARDS [15]. Compared with that case, our patient similarly developed shock, thrombocytopenia, renal dysfunction, and ARDS requiring ventilation, but he also had prominent meningeal signs and early AKI that improved with resuscitation. The absence or presence of later immune complications, such as arthritis or delayed thrombocytopenia, should be monitored during follow‐up.

Another comparable pediatric case was reported by Umer et al., describing an 11‐year‐old boy with fulminant meningococcemia and DIC who presented with high‐grade fever, purpuric rash, petechiae, ecchymosis, and circulatory collapse [16]. That patient recovered after timely antibiotics, aggressive fluid resuscitation, intensive care monitoring, and multidisciplinary management [16]. Our case is similar in age, initial shock, rash morphology, thrombocytopenia, and coagulopathy. However, our patient's course was more complicated because he developed ARDS requiring mechanical ventilation, severe initial AKI, elevated cardiac biomarkers, and required transfer to a tertiary pediatric ICU. This comparison emphasizes that early recognition and stabilization may improve survival, but serious organ complications can still evolve despite appropriate early therapy.

Horvatić et al. described a young child with fulminant meningococcal sepsis in whom death was attributed to circulatory collapse with DIC, adrenal involvement, and ARDS; autopsy showed lung edema, hyaline membranes, alveolar injury, and microthrombi in the skin and internal organs [17]. This is relevant to our case because it supports the pathophysiologic link between meningococcal septic shock, DIC, microvascular injury, and ARDS. Unlike that fatal case, our patient was recognized as critically ill at presentation, admitted to the ICU, started on broad‐spectrum antimicrobials, received hemodynamic support, and was referred for tertiary pediatric intensive care. With ongoing intensive supportive care, his respiratory status gradually improved, and he was successfully weaned from mechanical ventilation.

The infectious diseases consultant recommended treatment for both meningococcemia and rickettsial disease, which was reasonable in a child with fever, rash, shock, thrombocytopenia, and multiorgan involvement when local epidemiology and clinical uncertainty supported this differential. The CDC recommends doxycycline as the treatment of choice for suspected rickettsial infections in adults and children of all ages, especially when a life‐threatening rickettsial disease is suspected, and emphasizes that treatment is most effective when started early [18].

This case has several important teaching points. First, a toxic child with fever, shock, and a petechial or purpuric rash should be treated immediately as possible meningococcemia, even before microbiologic confirmation. Second, lumbar puncture may be unsafe in the early phase when shock, thrombocytopenia, or coagulopathy are present; therefore, deferring lumbar puncture in this patient was appropriate. Third, suspected meningococcal sepsis should be viewed as a systemic endothelial and inflammatory disease rather than only a bloodstream infection, as it may rapidly involve the coagulation system, kidneys, myocardium, lungs, and central nervous system. Fourth, early hemodynamic improvement does not exclude later organ deterioration, as demonstrated by the subsequent development of respiratory failure and ARDS after partial correction of shock and metabolic acidosis.

The main limitation of this case is the absence of microbiologic confirmation. Blood cultures were negative at both hospitals, and lumbar puncture was not performed because of clinical instability and coagulopathy. Another limitation is that the exact contribution of alternative diagnoses, including rickettsial infection or severe bacterial sepsis from another source, could not be fully excluded. Despite these limitations, the case remains educational because it demonstrates a classic and rapidly progressive syndrome of fever, meningeal signs, shock, hemorrhagic rash, coagulopathy, acute kidney injury, transient sepsis‐related myocardial stress, and ARDS, followed by respiratory improvement and successful weaning from mechanical ventilation after intensive supportive care.

## 6 Conclusion

The fulminant course of suspected meningococcal sepsis should be recognized when young patients present with fever, a petechial‐purpuric rash, and signs of circulatory collapse. Although prompt microbiologic confirmation may not always be possible, empirical antimicrobial therapy, aggressive fluid resuscitation, vasopressor support, infection control precautions, and prompt transfer to pediatric intensive care are key considerations. Despite the risk of rapid clinical deterioration, severe multiorgan dysfunction, and respiratory failure, multidisciplinary supportive care can lead to recovery, as demonstrated by renal recovery, successful weaning from mechanical ventilation, and discharge in good general condition. Follow‐up is important to assess for delayed dermatologic, neurologic, renal, cardiac, and psychological sequelae.

## Author Contributions

**Amr Khaled:** investigation, data curation, resources, writing – review and editing. **Mosub Qatu:** investigation, data curation, resources, writing – review and editing. **Noor Nabresi:** conceptualization, project administration, supervision, writing – review and editing. **Mahfouth Jallad:** investigation, resources, writing – review and editing. **Adil Alsweis:** conceptualization, data curation, visualization, writing – original draft, writing – review and editing. **Shatha Omar:** conceptualization, investigation, data curation, writing – original draft, writing – review and editing. **Yahya Qahwash:** investigation, resources, writing – review and editing.

## Funding

The authors have nothing to report.

## Disclosure

All the authors declare that the information provided here is accurate to the best of our knowledge.

## Consent

In accordance with ethical and legal guidelines, the patient's parents gave written informed consent for the publication of this case.

## Conflicts of Interest

The authors declare no conflicts of interest.

## Data Availability

The data that support the findings of this study are available on request from the corresponding author. The data are not publicly available due to privacy or ethical restrictions.

## References

[1] K. Avgoulea , G. Chronopoulou , A. Xirogianni , et al., “Two Cases of Rare Manifestations due to *Neisseria meningitidis* During the Post‐COVID‐19 Era in Greece,” Microorganisms 13, no. 9 (2025): 2071, 10.3390/microorganisms13092071. 41011403 PMC12472677

[2] J. Presa , L. Serra , C. Weil‐Olivier , and L. York , “Preventing Invasive Meningococcal Disease in Early Infancy,” Human Vaccines & Immunotherapeutics 18, no. 5 (2022): 1979846, 10.1080/21645515.2021.1979846. 35482946 PMC9196819

[3] M. Kheir , T. Catherine , W. Olivier , and S. Leng , “Meningococcal Disease in Older Adults: Challenges in Diagnosis and Management,” Infectious Disease and Therapy 15, no. 2 (2026): 443–459, 10.1007/s40121-025-01281-5. PMC12855718 41422470

[4] U. Al‐Anbagi , M. K. Ahmad , M. G. Mohamedali , et al., “Meningococcal Purpura Fulminans: A Rare Presentation in an Adult Case of Serogroup W135 Infection,” Cureus 17, no. 10 (2025): e94253, 10.7759/cureus.94253. 41216110 PMC12596455

[5] S. Taha , A. E. Deghmane , and M. K. Taha , “Recent Increase in Atypical Presentations of Invasive Meningococcal Disease in France,” BMC Infectious Diseases 24 (2024): 640, 10.1186/s12879-024-09547-y. 38926823 PMC11200843

[6] Centers for Disease Control and Prevention , “Chapter 8: Meningococcal Disease,” in Manual for the Surveillance of Vaccine‐Preventable Diseases (CDC, 2024), https://www.cdc.gov/surv‐manual/php/table‐of‐contents/chapter‐8‐meningococcal‐disease.html.

[7] Centers for Disease Control and Prevention , Clinical Guidance for Meningococcal Disease (CDC, 2026), https://www.cdc.gov/meningococcal/hcp/clinical‐guidance/index.html.

[8] The Royal Children's Hospital Melbourne , Clinical Practice Guidelines: Lumbar Puncture (Royal Children's Hospital Melbourne, 2024), https://www.rch.org.au/clinicalguide/guideline_index/Lumbar_puncture/.

[9] The Royal Children's Hospital Melbourne , Clinical Practice Guidelines: Meningitis and Encephalitis (Royal Children's Hospital Melbourne, 2024), https://www.rch.org.au/clinicalguide/guideline_index/Meningitis_encephalitis/.

[10] Centers for Disease Control and Prevention , Best Practices for Diagnosis of Haemophilus influenzae and *Neisseria meningitidis* Disease (CDC, 2026), https://www.cdc.gov/meningococcal/php/guidance/index.html.

[11] M. E. Colling and P. K. Bendapudi , “Purpura Fulminans: Mechanism and Management of Dysregulated Hemostasis,” Transfusion Medicine Reviews 32, no. 2 (2018): 69–76, 10.1016/j.tmrv.2017.10.001. 29157918

[12] C. Kennedy , S. Khilji , A. Dorman , and J. Walshe , “Bilateral Renal Cortical Necrosis in Meningococcal Meningitis,” Case Reports in Nephrology 2011 (2011): 274341, 10.1155/2011/274341. 24527235 PMC3914121

[13] A. Jain , J. Sankar , A. Anubhuti , D. K. Yadav , R. Lodha , and S. K. Kabra , “Prevalence and Outcome of Sepsis‐Induced Myocardial Dysfunction in Children With Sepsis With and Without Shock: A Prospective Observational Study,” Indian Pediatrics 55 (2018): 41–46. 29304220 10.1093/tropej/fmx105

[14] N. Yehya and N. J. Thomas , “Sepsis and Pediatric Acute Respiratory Distress Syndrome,” Journal of Pediatric Intensive Care 7, no. 2 (2018): 72–78. 10.1055/s-0038-1676133 PMC6506676 31073506

[15] A. Ghosh , N. Ganguly , P. Pal , and P. P. Giri , “Meningococcemia With ARDS, Reactive Arthritis and Immune Thrombocytopenia,” Pediatric Infectious Disease 5, no. 1 (2013): 19–21, 10.1016/j.pid.2012.12.013.

[16] H. Umer , M. Adil , and H. Jamil , “A Rare Case of Meningococcemia With Disseminated Intravascular Coagulation in an 11‐Year‐Old Boy,” Medical & Clinical Case Reports Journal 3, no. 3 (2025): 1270.

[17] E. Horvatić , I. Soldo , A. Vcev , et al., “Fulminant Meningococcal Sepsis in a Young Child: A Case Report,” Collegium Antropologicum 34, no. 4 (2010): 1461–1465. 21874740

[18] Centers for Disease Control and Prevention , Clinical Care of Other Spotted Fever Rickettsioses (CDC, 2025), https://www.cdc.gov/other‐spotted‐fever/hcp/clinical‐care/index.html.

